# Feasibility and Safety of Transcranial Direct Current Stimulation in an Outpatient Rehabilitation Setting After Stroke

**DOI:** 10.3390/brainsci10100719

**Published:** 2020-10-09

**Authors:** Brice T. Cleland, Melissa Galick, Amy Huckstep, Laura Lenhart, Sangeetha Madhavan

**Affiliations:** 1 Brain Plasticity Lab, Department of Physical Therapy, College of Applied Health Sciences, University of Illinois at Chicago, Chicago, IL 60612, USA; bcleland@uic.edu; 2 Shirley Ryan AbilityLab DayRehab Center, Homewood, IL 60430, USA; mgalick@sralab.org (M.G.); ahuckstep@sralab.org (A.H.); llenhart@sralab.org (L.L.)

**Keywords:** day care, medical, equipment safety, feasibility studies, neuroplasticity, stroke rehabilitation, transcranial direct current stimulation

## Abstract

Transcranial direct current stimulation (tDCS) has strong potential for outpatient clinical use, but feasibility and safety of tDCS has only been evaluated in laboratory and inpatient clinical settings. The objective of this study was to assess feasibility and safety of tDCS for stroke in an outpatient clinical setting. Individuals with stroke in outpatient therapy received tDCS during physical therapy sessions. Feasibility was assessed with screening, enrollment, withdrawal, and adherence numbers, tDCS impressions, and perceived benefits and detriments of tDCS. Acute changes in fatigue and self-reported function and pre-post changes in fatigue were also assessed. Safety was assessed as adverse events and side effects. In total, 85 individuals were screened, and 10 were enrolled. Most exclusions were unrelated to clinical feasibility. In total, 3 participants withdrew, so 7 participants completed 2 sessions/week for 5–6 weeks with 100% adherence. In total, 71% reported positive impressions of tDCS. tDCS setup decreased to 5–7 min at end of study. There was one adverse event unrelated to tDCS. Mild to moderate side effects (tingling, itching, pinching, and fatigue) were experienced. In total, 86% of participants recounted benefits of tDCS. There were acute improvements in function and energy. Results support the feasibility and safety of tDCS in an outpatient clinical setting.

## 1. Introduction

Stroke affects ~800,000 people each year in the United States and is a major cause of long-term disability [1,2]. Individuals with stroke experience impairments in the upper extremity (e.g., grip strength, dexterity, and motor control) and the lower extremity (e.g., balance, walking, and mobility) which limit activities of daily living [3]. The impact of stroke rehabilitation on impairment varies, with many individuals not regaining optimal function [4,5]. One potential explanation for why stroke rehabilitation may have a limited impact on long-term disability is the interhemispheric competition model [6]. This model describes an imbalance in interhemispheric inhibition excitability that may limit motor output and recovery on the more affected side [7].

To address hemispheric imbalance and promote recovery, recent investigations have used neuromodulation in combination with motor training to increase the excitability of the lesioned hemisphere and/or decrease the excitability of the non-lesioned hemisphere. One of the most commonly used modalities is transcranial direct current stimulation (tDCS), which involves the application of non-invasive low intensity direct currents to the scalp, altering the membrane potentials that affect neuronal excitability in the underlying cortical tissue [8]. Both tDCS and motor training can yield long-term effects through mechanisms similar to long-term potentiation and long-term depression. The combination of both interventions may yield greater neuroplasticity and functional improvement than either alone [8,9]. A large body of studies has elucidated the neurophysiological basis of tDCS and demonstrated facilitation of cognitive and motor processing and learning in the healthy and damaged brain [10,11,12]. Promising results with post-stroke motor rehabilitation have advanced treatment with tDCS to Class I level for the upper extremity [13]. In the lower extremity, tDCS can enhance strength, motor control, mobility, and balance [14,15,16,17,18,19] and may enhance improvements in walking speed and endurance when paired with gait training [20,21].

tDCS also appears to be safe, easy to use, and cost-effective. A recent review of data from 18,000 research sessions found no evidence of serious adverse events and found that tDCS does not cause neuronal damage at levels used in research in a variety of populations [22]. There is also considerable support for the safety of tDCS in the post-stroke population [23] and for the safety of home-based tDCS [24,25]. tDCS is relatively easy to use, as exemplified by the feasibility of home-based applications in multiple populations, including stroke [24,25,26]. Finally, tDCS units are small and cost as little as a few hundred dollars.

In a research setting, tDCS appears to be efficacious and safe. Considering that tDCS is also tolerable, easy to use, portable, and cost-effective, it has strong potential for use in a clinical setting [13]. Surprisingly, despite the large number of tDCS studies conducted in the laboratory, there is a dearth of studies that have been done in a clinical setting. Two studies have evaluated feasibility and safety of tDCS in an inpatient clinical setting in acute stroke [18,27]. However, to our knowledge, there have been no investigations into the safety and feasibility of tDCS in an outpatient clinical setting. Safety and feasibility in laboratory and inpatient settings may not translate to the outpatient clinical setting because of differences in environmental control and factors affecting clinician and patient attitudes and acceptability of a research modality. Hence, the objective of this exploratory study was to determine the feasibility and safety of using tDCS in an outpatient clinical setting.

## 2. Materials and Methods

### 2.1. Participants

All participants were recruited from the patient pool at the Shirley Ryan AbilityLab Homewood DayRehab Center, Homewood, IL, USA. Participants were enrolled if they were 18–80 years old, had a clinical history of single unilateral stroke and were scheduled to receive at least 6 weeks of gait training after the commencement of the study. Participants were excluded if they had contraindications to tDCS, including: history of epilepsy or seizures, use of a pacemaker, skin hypersensitivity, contact dermatitis, allodynia and/or hyperalgesia, or any other skin or scalp condition that could potentially be aggravated by tDCS. Participants were also excluded if they were pregnant, had neurological conditions besides stroke, or were unable to perform at least 10 min of walking on a treadmill or stepping on a recumbent stepper. This study was conducted in accordance with the Declaration of Helsinki, the protocol was approved by the Northwestern University IRB (STU00210329), and all participants provided written informed consent. Information such as date, location, and type of stroke, tests of physical function, and activities performed during rehabilitative treatment were extracted from patient records. Deidentified data that underlie study results will be shared by the corresponding author upon reasonable request from qualified investigators.

### 2.2. Clinical Setting

All participants received ongoing rehabilitative treatment at the Homewood DayRehab Center (part of the Shirley Ryan AbilityLab, Chicago, IL, USA), an outpatient neurorehabilitation center that provides therapy services to ~80 patients per day. Typically, patients undergo treatment 2–5 days per week for 3 h (half day) or 6 h (full day). Each day is split up into 55-min sessions, and each session focuses on one discipline: physical therapy, occupational therapy, or speech therapy. The specific daily and weekly schedule is individualized for each patient.

### 2.3. tDCS Training and Administration

Prior to the start of the study, physical therapist clinicians received ~5 h of instruction, training, and practice in the application of tDCS from research scientists with extensive experience in the use of tDCS. Training included a didactic lecture on the scientific premise and physiological basis of tDCS and hands-on activities to ensure proper landmark identification, electrode application, current administration, and troubleshooting. Training was provided to 5 clinicians, of whom 3 were ultimately involved in study procedures. The other 2 clinicians relocated or had other professional obligations.

tDCS was performed twice per week during a physical therapy session that focused on gait training. The application of tDCS was incorporated into the standard time for the treatment session (55 min) and applied during the ~15-min warmup exercise performed at each participant’s comfortable intensity and personalized based on each patient’s baseline self-selected gait speed. Warmup exercise was performed on a recumbent stepper (gait speed ≤0.4 m/s) or a treadmill (gait speed >0.4 m/s). To prepare for tDCS, the vertex was identified as the intersection of the midline from the nasion to the inion and the midline from tragus to tragus. The vertex was marked with indelible marker. The leg representation of the ipsilesional motor cortex was approximated as 1 cm posterior and 1 cm lateral from the vertex and marked with indelible marker [28]. Saline-soaked sponge electrodes (~5 × 5 cm; Caputron, New York City, NY, USA) were placed over the leg representation of the ipsilesional motor cortex (anode) and the contralesional supraorbital region (cathode). Electrodes were secured with an elastic wrap or headband. The electrodes used in this study are designed for multiple use, and each participant used their own pair of electrodes for all sessions. Anodal tDCS was applied with a constant current stimulator, Chattanooga Ionto (DJO Global, Lewisville, TX, USA). After a 30-s ramp-up, 1 mA current was applied for 15 min. The current density was 0.04 mA/cm^2^, and the total charge was 0.036 C/cm^2^, which were within safety limits [9]. tDCS duration was selected to be within the safety parameters used in previous studies in individuals with stroke [8,29]. tDCS was applied during warmup exercise because applying tDCS prior to motor training may optimize neuroplasticity [29,30].

The Chattanooga Ionto was selected for this study because it allows easy application of tDCS and is low-cost, portable, and adaptable. Users can apply between 0.5 and 4 mA of constant current (regardless of skin impedance) with an automatic 30-s ramp up and down to two different sites simultaneously. The device is available for USD ~500 and is pocket-sized (dimensions: 6 × 2 × 9 cm; mass: <1 kg). Users can interface with the device with a variety of reusable or single-use electrode types.

After warmup, tDCS equipment was turned off and removed, and high-intensity treadmill walking or overground gait training were performed. For high-intensity exercise, intensity was constantly modulated with the goal of obtaining a Rating of Perceived Exertion (RPE; Borg 0–10 scale [31]) between 6 and 8. Intensity was modified by increasing speed, increasing treadmill incline, decreasing external support, decreasing the number and duration of rest breaks, and adding ankle weights. To characterize warmup and high-intensity exercise, duration, speed, and RPE were recorded.

### 2.4. Feasibility

The number of patients screened and enrolled for the study was recorded, along with reasons for enrollment refusal and study withdrawal. For each participant, the number of sessions and weeks completed and adherence to the use of tDCS was recorded. To assess acute (within-session) changes in fatigue and function, participants completed the Rating-of-Fatigue Scale (ROF; a maximal rating of 10 represents total fatigue and exhaustion) [32] and the Patient-Specific Functional Scale (PSFS; a maximal rating of 10 represents no functional limitation) [33] before and after the application of tDCS. To assess pre-post changes in fatigue, participants completed the visual analogue fatigue scale (VAS-F) [34] before the first and after the last tDCS session. After the last tDCS session, participants completed a survey assessing their attitudes and impressions about tDCS, its feasibility and benefits, and barriers to use of this intervention (Appendix A). Impressions, perceptions, and suggestions were recorded from the study clinicians via interview.

### 2.5. Safety

The number of adverse effects associated with the application of tDCS was actively monitored during the study. In addition, any participant who reported intolerable discomfort or pain related to the application of tDCS or any other study procedures was planned to be removed from the study. Withdrawals and adverse effects were recorded. After completing the last session involving tDCS, participants completed a survey assessing tDCS-related side effects (Appendix B), largely based on work by Fertonani, Ferrari, and Miniussi [35].

### 2.6. Statistics

Related-samples Wilcoxon signed-rank tests were performed to compare ROF, PSFS, and VAS-F measures from the start to end of the tDCS session (ROF, PSFS) and from pre to post study (VAS-F). Statistical analyses were performed with IBM SPSS Statistics 22. A *p*-value of 0.05 was accepted as significant.

## 3. Results

### 3.1. Feasibility and Training Details

**Figure 1:**
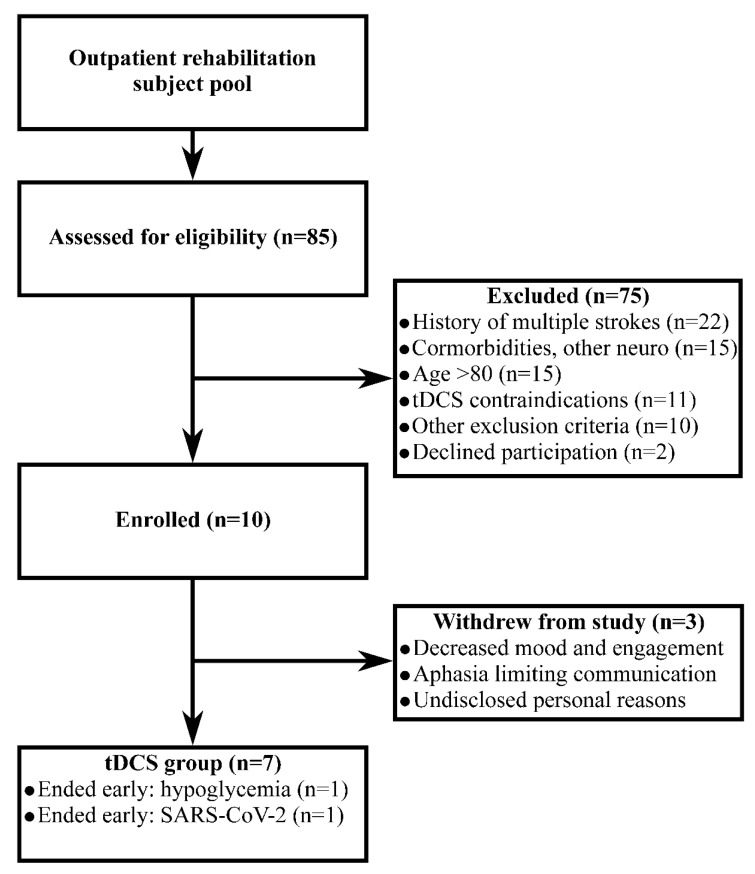
Flow chart.

In total, 85 individuals were screened between November 2019 to February 2020 for participation in this study after referral to the clinic with a diagnosis of stroke and/or recommendation for study consideration by their treating therapist. Most of these (69%) failed screening because they had history of multiple strokes, another neurological condition, or were greater than 80 years old. Screening identified 10 eligible individuals with stroke who were recruited, provided informed consent, and started the study. Of these, 3 participants withdrew from the study after enrollment because: (1) decreased mood that limited accurate feedback and engagement, (2) aphasia prevented accurate feedback to clinicians, and (3) other undisclosed personal reasons. Thus, data from 7 participants who received tDCS were included in the final analyses. A flow chart is shown in Figure 1, and demographics are presented in Table 1. Comorbidities included: hypertension (n = 6), type II diabetes (n = 4), cardiac surgery (n = 1), atrial fibrillation (n = 1), obesity (n = 1), and chronic tobacco use (n = 1).

Participants completed 10–12 sessions distributed as 2 sessions/week for 5–6 weeks. Attendance and transcranial direct current stimulation (tDCS) application rate for scheduled sessions were 100%. In two participants, sessions were completed for 5 instead of 6 weeks. One participant was removed from the study because of a hypoglycemic event (see Safety section below); the study was discontinued in another participant because of the severe acute respiratory syndrome coronavirus 2 (SARS-CoV-2, COVID-19) pandemic. Across all participants and sessions, mean (standard deviation) warmup duration was 15.1 (0.2) min and high intensity training duration was 18.2 (1.7) min. Warmup walking/stepping speed was 0.35 (0.22) m/s, and high intensity training speed was 0.55 (0.25) m/s. Warmup RPE was 4.2 (1.1), and high intensity training RPE was 6.3 (1.1) on a scale of 10. Average ROF (Z = −2.4, *p* = 0.02) and average PSFS (Z = −2.4, *p* = 0.02) increased from the start to the end of each exercise session (Table 2). VAS-F energy rating increased from before the first tDCS session to after the last tDCS session (Table 2; Z = −2.0, *p* = 0.04). There was no change in VAS-F fatigue or total rating (*p* ≥ 0.61).

**Figure 2:**
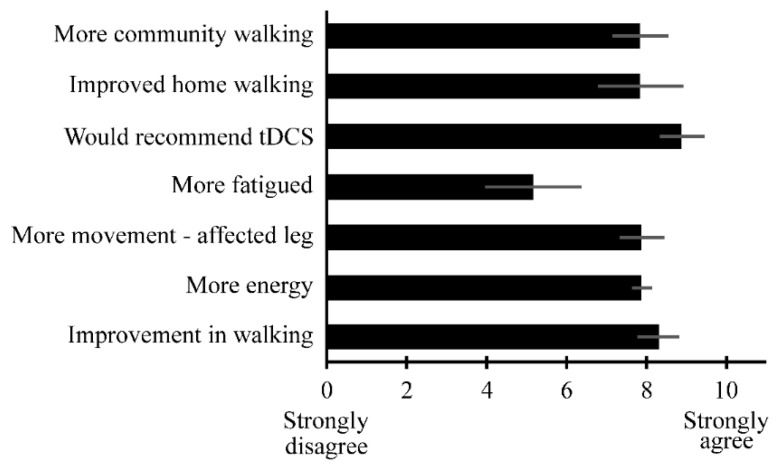
Perceived benefits and detriments of tDCS. Ratings are from a 0–10 scale. All questions assessed potential benefits of tDCS, except for feeling “more fatigued,” which assessed a potential detriment of tDCS. See Appendix A for questions. Bars are average, and error bars are standard error.

In the post-study survey about tDCS, participants reported that they prospectively had expected tDCS to benefit their walking (n = 2), brain (n = 2), flexibility (n = 1), talking (n = 1), legs (n = 1), and strength (n = 1). Retrospective, self-reported benefits of tDCS were improvements in walking (n = 3), community walking (n = 1), energy (n = 1), balance (n = 1), standing (n = 1), dressing (n = 1), overall physical function (n = 1), back (n = 1), and arm (n = 1). On average, participants gave an agreement rating of ≥7.8/10 for all perceived benefits of tDCS and gave an agreement rating of 5.1/10 for the perceived detriment of more fatigue (Figure 2). In total, 5 participants reported positive impressions of tDCS, 1 reported some worries about potential side effects, and 1 did not convey their impressions. Aspects that participants reported liking about tDCS included: perceived improvements in walking (n = 4), the short duration of the intervention (n = 1), the convenience of the location (n = 1), and the skill of study clinicians (n = 1). In total, 5 participants indicated that they liked the frequency of tDCS, while 2 indicated that they would have liked a greater frequency. Only two dislikes were noted: the comfort of the headbands used (n = 1) and the overall comfort of tDCS (n = 1). The only suggested changes were to improve the comfort of the headbands (n = 1) and to increase the frequency of tDCS (n = 1).

Clinicians involved in the study reported that the tDCS setup time decreased from 10 min at the start of the study to 5–7 min at the end of the study. Setup was identified as a task that could be completed by rehab technicians. Clinicians noted that because priming took 15 min, participants were limited to 20–25 min of high intensity gait training. The study clinicians also suggested that it may be beneficial to do some of the high intensity training on a recumbent stepper in combination with tDCS. Finally, the clinicians subjectively noted that participants receiving tDCS had an increased awareness, commitment, and investment in their treatment plan of care.

### 3.2. Safety

Of the 7 participants, one was removed from the study because of an adverse event. In this individual, the 10th session was stopped during high intensity treadmill walking because the participant had a hypoglycemic event (blood glucose = 60 mg/dL). The participant was transferred to a supine position with lower extremity elevated, and orange juice and a granola bar were provided. The participant’s family reported that the participant had experienced high blood sugar prior to the treatment session, and insulin had been administered. This participant continued with rehabilitative treatment but was withdrawn from the study.

In the post-study survey of tDCS-related side effects, five participants reported mild to moderate side effects from tDCS. Reported side effects were mild to moderate tingling (n = 3), mild itching (n = 2), mild to moderate fatigue (n = 2), and mild pinching (n = 1). Focal side effects were felt in the vicinity of the tDCS electrodes. Side effects were reported at the beginning (n = 2), middle (n = 1), and end (n = 1) of the stimulation, or throughout the entirety of stimulation (n = 1). No participants reported that side effects affected their performance, and no pain was reported.

## 4. Discussion

In this preliminary study, we found evidence that tDCS is feasible and safe for persons with stroke with other co-morbidities when applied in an outpatient clinical setting. Impressions of tDCS were positive, and participants completed the tDCS sessions without any adverse events. Self-reported benefits of the treatment provide preliminary support for the efficacy of tDCS applied in a clinical setting.

### 4.1. Feasibility

Our results support the feasibility of applying tDCS in an outpatient clinical setting. During the eligibility assessment, most potential participants were excluded for reasons related to internal validity (e.g., multiple strokes, other neurological conditions, or age), not because of contraindications to tDCS. Overall, 85% (72 out of 85) of the individuals who were screened could have been included in the study if not for these internal validity controls. It is likely that it would be safe for a large portion of the outpatient rehabilitation clinical population to receive tDCS as part of their plan of care [23]. Of the three participants who withdrew after enrolling in the study, one withdrew because they could not communicate well enough to complete study assessments (aphasia). This likely would not preclude the application of tDCS under normal clinical circumstances. Considering a 20% withdrawal rate (based on 2 out of 10 enrolled participants), we estimate that 68% of the clinical population (58 out of 85) could have received tDCS. These results suggest that it is likely that tDCS would be feasible for a large portion of the outpatient clinical population to receive tDCS as part of their plan of care after stroke. This conclusion is in accordance with studies suggesting the feasibility of inpatient tDCS application in acute stroke [18,27]. Additionally, it may be feasible for even more of the clinical population to receive tDCS if some unnecessary traditional exclusion criteria are omitted [36].

Participants who received tDCS tolerated the treatment well and attended all sessions. Response to warmup and high intensity exercise appeared to be normal. We found evidence of acute reductions in perceived physical function limitation and pre-post improvements in perceived energy. If tDCS improves patient energy levels, this may provide an added benefit for individuals experiencing low energy levels, especially after stroke. However, it is interesting to note that fatigue symptoms did not change from pre to post study. Although fatigue is a common acute side effect of tDCS [23,37], and our participants reported acute increases in fatigue (ROF), our findings suggest that these changes in fatigue are not long-term. Furthermore, tDCS may reduce fatigue in populations with chronic fatigue (e.g., multiple sclerosis, fibromyalgia, and chronic fatigue syndrome) [38], but our study does not support a role of tDCS for fatigue reduction in the post-stroke population.

Six out of seven (86%) participants recounted perceived benefits of tDCS, including improved walking. This coincides with a number of publications demonstrating the potential benefits of tDCS for lower limb movement [14,15,16,17,18,19,20,21,39]. Furthermore, five out of seven (71%) participants reported positive impressions of tDCS and that they had expected to benefit from the treatment in a variety of ways. Five out of seven (71%) participants indicated that they liked the frequency of tDCS, and four out of seven (57%) liked the perceived improvements in walking. In fact, two of our participants indicated that they would have liked tDCS for a longer duration. Only two out of seven (29%) participants reported minor issues with the comfort of the treatment (tDCS overall and headbands). Issues with the comfort of headbands or elastic wrap used to secure tDCS electrodes may be addressed with single-use adhesive electrodes. This approach would likely reduce setup time and discomfort, although the adhesive may not be strong enough to hold throughout exercise. In this study, we chose reusable sponge electrodes secured with headbands and elastic wrap because this approach is less expensive, which may be desired in a clinical setting. Our findings suggest that clinicians should be able to rapidly establish patient support for the use of tDCS in their treatment regimen, as seen in home-based tDCS applications after stroke [25]. This is supported by clinicians’ report of increased participant awareness, commitment, and investment in their treatment plan of care.

Clinicians were eager to use tDCS (based on previous knowledge regarding the priming effects on motor recovery), and the two clinicians who did not participate did so for non-tDCS related reasons. Required setup time decreased with experience, eventually only requiring 5–7 min, which can be easily incorporated into the standard treatment. As noted, setup could be performed by rehab technicians to decrease clinician burden and maximize time spent on skilled intervention. As found in this study, greater use of tDCS by clinicians will likely provide insights into ways to optimize this treatment in the outpatient clinical setting.

### 4.2. Safety

There was one adverse event that took place during this study, which appeared to be unrelated to the administration of tDCS. Study participants noted mild to moderate side effects from tDCS, including tingling, itching, pinching, and fatigue. Most of these side effects were only experienced during current ramp up or ramp down. Consistent with our findings, some of the most commonly reported side effects in the healthy and post-stroke population are tingling and itching [23,37]. It is also important to note that no participants reported that side effects affected their performance, no pain was reported, and symptoms were mild enough that participants chose to continue with the treatment sessions. Overall, these findings are consistent with previous research supporting the safety of tDCS [22,23]. However, researchers need to be diligent about checking contraindications to tDCS prior to application [40].

### 4.3. Limitations

Our findings provide preliminary support for feasibility and safety of tDCS in an outpatient clinical setting, but our conclusions are limited by several factors. Our sample size was small (n = 7), which may have limited interpretations of our results. However, our sample size is consistent with other demonstrations of tDCS feasibility in stroke in other settings [25,27]. Although our results support feasibility, there are limitations to immediate clinical implementation of tDCS. One limitation is that there is no insurance coverage for tDCS currently. Thus, use of tDCS may require out-of-pocket coverage. Another limitation is that, although tDCS devices are widely available, the safety of these devices has not been determined, and clear regulation is not present. For example, common iontophoresis devices do not have safety limits on current output and are not designed for application to the scalp [41]. Finally, the study clinicians noted that because the tDCS time (15 min) was longer than typical warmup exercise, the amount of high intensity training was slightly decreased. In the future, a more effective strategy may be to gradually increase the intensity of exercise throughout the application of tDCS so therapy time is not affected. For example, after 3–5 min of low-intensity exercise, the intensity can be increased throughout the remainder of tDCS.

## 5. Conclusions

In this study, we found preliminary evidence that the use of tDCS in an outpatient clinical setting is feasible and safe. Further research is needed to determine the efficacy of this treatment, and insights from clinicians can help optimize application of tDCS in the clinic.

## Figures and Tables

**Table 1:** Participant demographics. Individual and group average data are shown. For categorical variables, group data shows count in each category; for continuous variables, values are mean (standard deviation). Short dashes represent data that was unavailable from patient records. M: male; F: female; C: Caucasian; AA: African American; MCA: middle cerebral artery; C: cortical; SC: subcortical; I: ischemic; H: hemorrhagic; L: left; R: right.

	Gender	Age (Years)	Race	Time Since Stroke (Months)	Stroke Location	Stroke Type	Paretic Limb
T1	M	55	C	3.6	frontoparietal	H	L
T2	M	67	AA	5.4	-	-	L
T3	M	57	AA	5.3	frontal, insula	I	R
T4	F	65	C	1.0	medulla	-	R
T5	M	64	C	4.0	pons	I	L
T6	M	54	AA	2.6	basal ganglia	H	L
T7	M	56	C	2.7	MCA	I	R
Group	M = 6	59.7 (5.4)	C = 4	3.5 (1.6)	C = 3	I = 3	L = 4
	F = 1		AA = 3		SC = 3	H = 2	R = 3

**Table 2:** Acute and pre-post effects of tDCS on fatigue and function. Averages for the Rating-of-Fatigue Scale (ROF; 10 point maximum) and Patient-Specific Functional Scale (PSFS; 10 point maximum) are shown from before and after each tDCS and exercise session. Visual analogue fatigue scale (VAS-F) values are shown from the start of the study to the end (5–6 weeks later). Values are mean (standard deviation). * *p* < 0.05 between pre and post.

**Acute Effects**		
	**Pre Session**	**Post Session**
ROF	2.6 (1.3)	6.2 (0.9) *
PSFS	4.3 (2.0)	5.3 (1.9) *
**Pre-Post Effects**		
	**Pre Study**	**Post Study**
VAS-F	71.8 (17.3)	70.8 (24.7)
fatigue	39.9 (26.1)	36.1 (23.8)
energy	25.3 (8.4)	30.8 (6.3) *

## Appendix A. tDCS Attitude and Impressions Participant Survey

(1) What are your impressions about transcranial direct current stimulation (tDCS)? Some examples of things to consider are length of time, intensity, side effects, perceived barriers and benefits).
(2) What did you like about tDCS?
(3) What did you dislike about tDCS?
(4) Is there anything about the tDCS treatment that you would change?
(5) Before you received tDCS, how did you hope to benefit from it?
(6) Do you feel that you benefited from tDCS? Please explain.
(7) Did you receive tDCS as frequently as you would have liked? Please explain. How often would you have ideally received tDCS?
(8) Did you experience any negative side effects from tDCS or the exercise with which it was paired?
(9) Is there anything you would change about the treatment using tDCS?

Please answer the following questions using the scale below:

1	2	3	4	5	6	7	8	9	10
Strongly disagree									Strongly agree

(1) I noticed an improvement in my walking following the use of tDCS.
(2) I felt like I had more energy following my tDCS session.
(3) I noticed more movement in my affected leg following tDCS.
(4) I was more fatigued after tDCS.
(5) I would recommend tDCS to other individuals who have had a stroke.
(6) I have noticed an improvement in my ability to walk around the home.
(7) I am walking more often out in the community.

## Appendix B. tDCS Side Effects Survey

(1) Did you experience any discomfort or annoyance during any session involving electrical stimulation? Please answer the following questions regarding the different sensations and indicate the degree of intensity of your discomfort according to the following scale:
  - None = I did not feel the described sensation (0)
  - Mild = I mildly felt the described sensation (1)
  - Moderate = I felt the described sensation (2)
  - Considerable = I felt the described sensation to a considerable degree (3)
  - Strong = I strongly felt the described sensation (4)

Itching:	□ None	□ Mild	□ Moderate	□ Considerable	□ Strong
Tingling:	□ None	□ Mild	□ Moderate	□ Considerable	□ Strong
Pain:	□ None	□ Mild	□ Moderate	□ Considerable	□ Strong
Burning:	□ None	□ Mild	□ Moderate	□ Considerable	□ Strong
Warmth/Heat:	□ None	□ Mild	□ Moderate	□ Considerable	□ Strong
Pinching:	□ None	□ Mild	□ Moderate	□ Considerable	□ Strong
Metallic/Iron taste:	□ None	□ Mild	□ Moderate	□ Considerable	□ Strong
Fatigue:	□ None	□ Mild	□ Moderate	□ Considerable	□ Strong
Headache:	□ None	□ Mild	□ Moderate	□ Considerable	□ Strong
Skin redness:	□ None	□ Mild	□ Moderate	□ Considerable	□ Strong
Other: __________	□ None	□ Mild	□ Moderate	□ Considerable	□ Strong

(2) When did any discomfort begin?

□ At the beginning of the stimulation

□ At approximately the middle of the stimulation

□ Towards the end of the stimulation

(3) How long did it last?

□ It stopped quickly

□ It stopped in the middle of the stimulation

□ It stopped at the end of the stimulation

(4) How much did these sensations affect your performance?

□ Not at all □ Slightly □ Considerably □ Much □ Very much

(5) Were these sensations located over the head or in a different location?

□ On the head ____________________________ □ Other____________________________

(6) If you experienced any pain during the electrical stimulation, please mark how much your pain was on this scale from 0 to 10.
(7) Do you have any other comments related to side effects of tDCS?
